# The relationship between intraoperative glucose levels and length of hospital stay in patients with a femoral neck fracture: a retrospective study based on the MIMIC-IV database

**DOI:** 10.3389/fsurg.2024.1476173

**Published:** 2024-11-20

**Authors:** Yan Ni, Cheng-ming Hu, Chao Li, Ting Zhang, Ying-xue Bao

**Affiliations:** ^1^ Nursing Department, Sir Run Run Shaw Hospital, Zhejiang University School of Medicine, Hangzhou, China; ^2^ Department of Operation Room, Zhejiang Provincial People’s Hospital (Affiliated People’s Hospital), Hangzhou Medical College, Hangzhou, Zhejiang, China

**Keywords:** femoral neck fracture, length of hospital stay, intraoperative glucose, influencing factors, medical information mart for intensive care database

## Abstract

**Objective:**

This retrospective study aimed to explore the relationship between intraoperative glucose (IG) and the length of hospital stay (LOS) in patients with femoral neck fractures via the Medical Information Mart for Intensive Care-IV (MIMIC-IV) database.

**Methods:**

A generalized additive model was performed to explore the relationship between IG levels and LOS. Restricted cubic spline curves were used to analyze the dose-response relationship between IG levels and prolonged LOS (or 7-day LOS). Threshold effect analysis was conducted to assess the key points influencing their association. Receiver operating characteristic (ROC) curve and decision curve analysis (DCA) were performed to evaluate the predictive performance of IG levels for LOS.

**Results:**

A total of 743 patients with femoral neck fractures were enrolled from the MIMIC-IV database. We found that there was a non-linear relationship between IG and the LOS (or prolonged LOS/>7 days LOS). Moreover, their relationship was still significant even after adjusting for potential confounders. The threshold effect showed that IG was significantly related to a prolonged LOS when it was >137 mg/dl, and IG was significantly related to a 7-day LOS when it was >163 mg/dl. ROC showed that IG had a better function in predicting a 7-day LOS in participants with IG >163 mg/dl than in predicting a prolonged LOS among participants with IG >137 mg/dl. Moreover, the DCA results showed that IG can obtain a favorable net benefit in clinical settings in predicting a 7-day LOS among participants with IG >163 mg/dl.

**Conclusions:**

In summary, there was a non-linear relationship between IG levels and LOS. In patients with IG levels >163 mg/dl, using IG content to predict an LOS >7 days had a good function.

## 1 Introduction

Hip fractures account for 14% of fractures in the elderly population, but consume approximately 72% of total healthcare expenditure, with projected medical costs expected to exceed $18.2 billion by 2025 (1). Among hip fractures, femoral neck fractures are the most typical and common type, predominantly affecting the elderly population, thereby imposing a significant burden on public healthcare systems and society. Currently, surgical intervention is the primary treatment for femoral neck fractures, requiring hospitalization. Studies have found that 54.90% of patients with femoral neck fractures experience a prolonged length of hospital stay (LOS) (2). A prolonged LOS increases the burden of clinical care and affects the equitable distribution of medical resources. It is also associated with higher medical costs, readmission rates, and mortality. Schneider et al. (3) reported that prolonged hospitalization in patients undergoing surgery for femoral neck fractures increases the risk of death in 30 days (OR = 2.500). Therefore, identifying factors influencing a prolonged LOS, implementing targeted early interventions, and effectively shortening LOS are essential for improving clinical outcomes, reducing healthcare costs and clinical care burden, and facilitating the rational allocation of medical resources.

Domestic reports indicate that 24.39% of orthopedic surgery patients experience perioperative stress-induced hyperglycemia, which affects LOS (4). As the incidence of blood sugar abnormalities increases annually, there is a risk of perioperative hyperglycemia in non-diabetic patients and those with undiagnosed diabetes. The “2020 Expert Consensus on Perioperative Blood Glucose Management” (5) recommends that perioperative blood glucose be controlled within the range of 5.5–10 mmol/L. However, there may be differences in blood sugar management among patients undergoing different surgeries. For patients undergoing surgery for femoral neck fractures, intraoperative blood sugar management needs to be strengthened, placing higher demands on operating room nursing staff. Previous studies on other diseases have found that perioperative hyperglycemia is associated with LOS in surgical patients, and intraoperative blood sugar levels should be kept below 180 mg/dl (6). However, research specifically targeting femoral neck fracture patients is limited, and there is a lack of quantitative assessment and analysis of the impact of intraoperative glucose (IG) levels on the hospitalization time of such patients.

This study analyzes the potential impact of IG levels on the hospitalization time of femoral neck fracture patients based on the Medical Information Mart for Intensive Care-IV (MIMIC-IV) database in the United States, aiming to provide an auxiliary reference for the formulation of targeted nursing decisions in the operating room.

## 2 Data and methods

### 2.1 Data source and study population

All the data in this study were collected from the MIMIC-IV database (https://mimic.mit.edu/iv/). The MIMIC-IV database is a large-scale, publicly available medical database that collected clinical data from over 300,000 patients admitted to the intensive care units (ICUs) of Beth Israel Deaconess Medical Center (BIDMC) between 2008 and 2019. It includes a vast amount of medical information such as demographics, laboratory tests, treatment processes, and more. MIMIC-IV has been approved by the Institutional Review Boards of the Massachusetts Institute of Technology and BIDMC, adheres to the principles of the Helsinki Declaration, and employs anonymization techniques to protect patient privacy, thus waiving the need for informed consent. The personnel responsible for data extraction for this study have completed training, passed the CITI program exam, and obtained permission to use the MIMIC-IV database.

**Figure 1:**
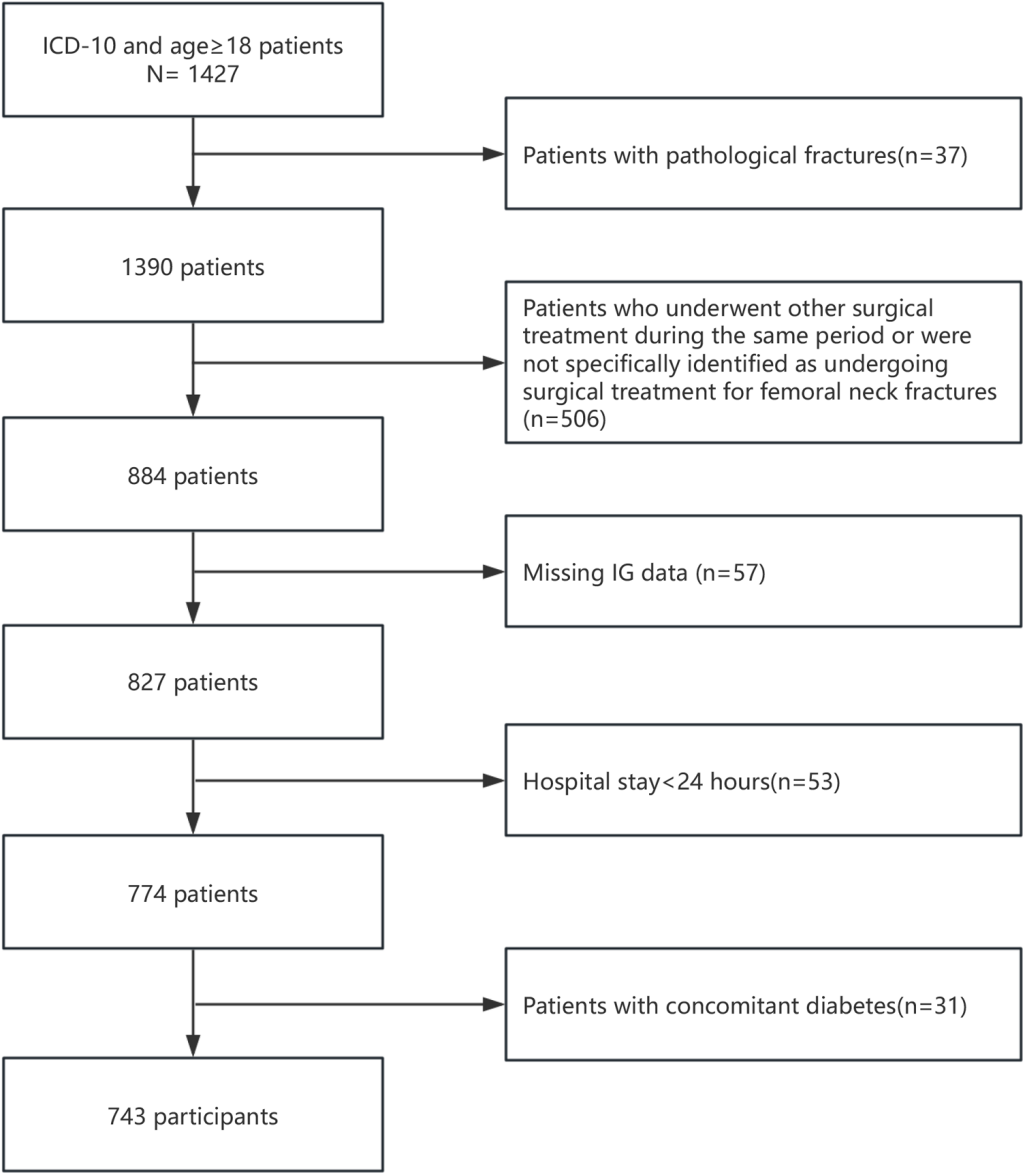
Flow charts of the inclusion and exclusion criteria for the patients.

The study population consisted of surgical patients diagnosed with femoral neck fractures in the MIMIC-IV database. Inclusion criteria are as follows: (1) Patients diagnosed with femoral neck fractures; (2) Age ≥18 years. Exclusion criteria are as follows: (1) Patients with pathological fractures; (2) Patients who underwent other surgical treatments during the same period or were not specifically identified as undergoing surgical treatment for femoral neck fractures; (3) Missing IG data; (4) Hospital stay <24 h; (5) Patients with concomitant diabetes. A flow chart of the inclusion and exclusion criteria of patients is shown in Figure 1.

### 2.2 Covariables and outcomes

The following data were extracted from the MIMIC-IV database using the PostgreSQL tool (version 12): (1) Demographic data including gender and age; (2) Physiological data including body mass index (BMI), systolic blood pressure, and diastolic blood pressure; (3) Disease data including fracture type and surgical procedure; (4) Personal history including smoking history and alcohol history; (5) Comorbidities including anemia, osteoporosis, hypertension, chronic obstructive pulmonary disease (COPD), and coronary artery disease; (6) Laboratory indicators including admission baseline anion gap, serum creatinine, serum potassium, total calcium, hemoglobin, mean corpuscular hemoglobin (MCH), mean corpuscular hemoglobin concentration (MCHC), and IG levels; (7) Clinical outcomes including admission to the ICU, LOS, and mortality.

Furthermore, a prolonged LOS and an LOS >7 days also served as outcomes for patients. A prolonged LOS was determined based on previous literature standards (7, 8), with the 75th percentile of the total hospital stay duration considered as a prolonged LOS.

### 2.3 Statistical analysis

All data were analyzed using SPSS 25.0 and RStudio software (version 4.1.2). Categorical variables were presented as [*n* (%)] and analyzed using the chi-square test. Normally distributed continuous variables were presented as mean ± standard deviation and compared between groups using the *t*-test. Non-normally distributed continuous variables were presented as median (P25, P75) and analyzed using the Mann–Whitney *U* test. The generalized additive model (GAM) was used to analyze the relationship between IG levels and LOS. GAM is an extension of the Generalized Linear Model (GLM) and is an additive modeling technique. GAM assumes that the relationship between individual predictor variables and the dependent variable follows a smooth pattern, which can be either linear or non-linear, and then predicts the outcome event by estimating the smooth relationships simultaneously. Restricted cubic spline (RCS) curves, a flexible method to simulate the complex non-linear relationship between variables, were used to verify the dose-response relationship between IG levels and a prolonged LOS (or an LOS >7 days). Threshold effect analysis was conducted to quantitatively assess the impact of IG levels on a prolonged LOS (or an LOS >7 days). Receiver operating characteristic (ROC) curve and decision curve analysis (DCA) were performed to evaluate the predictive performance of IG levels for a prolonged LOS (or an LOS >7 days). In the DCA plot, two reference lines are included for comparison: (1) the “None” net benefit line assumes that no patients receive intervention, and the net benefit is zero; (2) the “All” net benefit line assumes that all patients receive intervention, and this line shows the net benefit in this extreme scenario. The net benefit curve demonstrates the model's net benefit at various threshold probabilities, compared to the reference lines. A *P*-value of <0.05 was considered statistically significant (with a significance level of *α* = 0.05, two-sided test).

## 3 Results

### 3.1 Baseline characteristics of the participants

A total of 743 patients with femoral neck fractures were included in the study, among which there were 272 men (36.61%) and 471 women (63.39%). The age range was 19–91 years, with a mean age of 74.79 ± 15.55 years. LOS ranged from 1.1 to 76.9 days, with an average of 6.08 days and a median of 4.8 (3.7–6.7) days. According to the criteria for a prolonged hospital stay, 185 out of the 743 patients with femoral neck fractures (24.90%) experienced a prolonged hospital stay. Clinical data analysis showed that there were statistically significant differences (*P* < 0.05) between the prolonged LOS group and the non-prolonged LOS group in terms of gender, surgical method, anemia, anion gap, blood creatinine, blood potassium, MCHC, and IG levels (Table 1).

**Table 1:** Baseline characteristics.

Variable	Non-prolonged LOS (*n* = 558)	Prolonged LOS (*n* = 185)	*χ*^2^/*Z*/*t*	*P*-value
BMI (kg/m^2^)	23.600 (21.100–27.100)	24.200 (21.600–27.500)	−1.323	0.186
Age (years)	79.000 (68.000–87.000)	77.000 (65.000–86.000)	1.149	0.250
Systolic blood pressure (mmHg)	122.000 (110.000–137.000)	120.000(111.000–130.000)	1.302	0.193
Diastolic blood pressure (mmHg)	70.000 (60.000–77.000)	70.000 (62.000–76.000)	−0.177	0.859
Anion gap (mEq/L)	13.000 (12.000–15.000)	14.000 (12.000–16.000)	−3.169	0.001
Serum creatinine (mg/dl)	0.900 (0.700–1.100)	1.000 (0.700–1.300)	−2.815	0.005
Serum potassium (mmol/L)	4.100 (3.800–4.400)	4.100 (3.800–4.600)	−2.102	0.035
Total calcium (mg/dl)	8.600 (8.200–9.000)	8.500 (8.200–8.900)	0.880	0.378
hemoglobin (g/dl)	11.437 ± 1.827	11.144 ± 1.962	1.854	0.064
MCH (pg)	30.600 (29.500–32.000)	30.900 (29.400–32.200)	−0.870	0.384
MCHC, %	33.200 (32.300–34.000)	33.100 (32.200–33.500)	2.212	0.027
IG (mg/dl)	118.000 (103.000–134.000)	128.000 (110.000–155.000)	−4.882	<0.001
Sex, *n* (%)				
Male	188 (33.692)	84 (45.405)	8.215	0.004
Female	370 (66.308)	101 (54.595)		
Fracture types, *n* (%)				
Closed fracture	548 (98.208)	183 (98.919)	0.442	0.506
Open unspecified	10 (1.792)	2 (1.081)		
Surgical approach, *n* (%)				
Total hip arthroplasty	109 (19.534)	44 (23.784)	19.821	<0.001
Half hip replacement	199 (35.663)	92 (49.730)		
Internal fixation	250 (44.803)	49 (26.486)		
Smoking, *n* (%)				
Yes	61 (10.932)	29 (15.676)	2.937	0.087
No	497 (89.068)	156 (84.324)		
Alcohol drinking, *n* (%)				
Yes	13 (2.330)	3 (1.622)	0.331	0.565
No	545 (97.670)	182 (98.378)		
Anemia, *n* (%)				
Yes	197 (35.305)	119 (64.324)	47.87	<0.001
No	361 (64.695)	66 (35.676)		
Osteoporosis, *n* (%)				
Yes	107 (19.176)	28 (15.135)	1.526	0.217
No	451 (80.824)	157 (84.865)		
Hypertension, *n* (%)				
Yes	270 (48.387)	80 (43.243)	1.475	0.224
No	288 (51.613)	105 (56.757)		
Chronic obstructive pulmonary disease, *n* (%)				
Yes	23 (4.122)	8 (4.324)	0.014	0.905
No	535 (95.878)	177 (95.676)		
Coronary heart disease, *n* (%)				
Yes	26 (4.659)	14 (7.568)	2.307	0.129
No	532 (95.341)	171 (92.432)		
ICU, *n* (%)				
No	524 (93.907)	125 (67.568)	87.223	<0.001
Yes	34 (6.093)	60 (32.432)		
Obesity, *n* (%)				
No	490 (87.814)	158 (85.405)	0.723	0.395
Yes	68(12.186)	27(14.595)		

BMI, body mass index; MCH, mean corpuscular hemoglobin; MCHC, corpuscular hemoglobin concentration; LOS, length of hospital stay; IG, intraoperative glucose; ICU, intraoperative glucose.

### 3.2 Relationship between the IG and LOS

**Figure 2:**
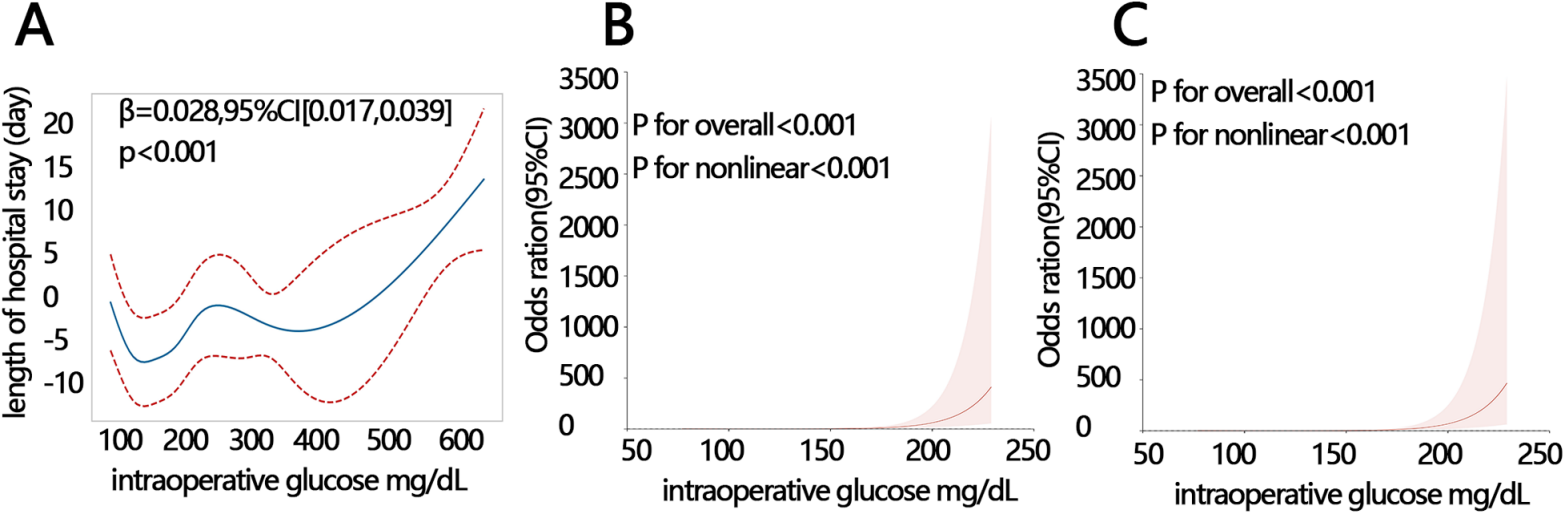
IG level was correlated with LOS. **(A)** The correlation between IG level and LOS by GAM. **(B)** The correlation between IG level and a prolonged length of hospital stay by RCS. **(C)** The correlation between IG level and an LOS >7 days by RCS. IG, intraoperative glucose; LOS, length of hospital stay; RCS, restricted cubic spline; GAM, generalized additive model.

The relationship between IG level and the length of the hospital stay was explored via GAM and RCS. The results of GAM showed that there was a non-linear relationship between them (Figure 2A, *P* < 0.001). Next, we verified their non-linear relationship according to the two groups’ outcomes via RCS by setting LOS as a categorical variable. One group was the prolonged LOS and non-prolonged LOS, and the other group was a LOS >7 days and a LOS <7 days. The RCS result showed a non-linear relationship between IG level and a prolonged LOS (Figure 2B, *P* for non-linear <0.001). The same correlation was found between IG level and a 7-day LOS (Figure 2C, *P* for non-linear <0.001). These results showed that there was a significantly non-linear relationship between IG level and LOS.

Therefore, we further performed the generalized linear regression model to explore the relationship between IG level and LOS. The results showed that IG level was significantly positively related to LOS. Their relationship was still significant even after adjusting for potential confounders in adjusted model 2 (adjusting for sex, ICU, surgical approach, and anemia) and model 3 (adjusting for anion gap, serum creatinine, MCHC, and serum potassium) (Table 2, all *P* < 0.01). IG level was also significantly associated with a prolonged LOS and an LOS >7 days in three other models (all *P* < 0.001).

**Table 2:** Generalized linear model of the relationship between IG level and LOS.

Outcome	LOS		Prolonged LOS		>7 days LOS	
Model	*β* (95% CI)	*P*-value	*β* (95% CI)	*P*-value	*β* (95% CI)	*P*-value
Model 1						
IG	0.028 (0.017–0.039)	<0.001	0.003 (0.002–0.004)	<0.001	0.003 (0.003–0.004)	<0.001
Model 2						
IG	0.015 (0.005–0.026)	0.004	0.002 (0.001–0.003)	<0.001	0.002 (0.002–0.003)	<0.001
Model 3						
IG	0.025 (0.015–0.036)	<0.001	0.003 (0.002–0.004)	<0.001	0.003 (0.003–0.004)	<0.001

IG, intraoperative glucose; LOS, length of hospital stay; CI, confidence interval.

Model 1: without adjustment. Model 2: adjusting for sex, ICU, surgical approach, and anemia. Model 3: adjusting for anion gap, serum creatinine, MCHC, and serum potassium.

Next, we further explored the relationship between them in detail via the threshold effect and disclosed related turning point (Table 3). The results showed that IG level was significantly related to a prolonged length of hospital stay when it was >137 mg/dl [*β* = 1.075, 95% confidence interval (CI): 1.043–1.108], and IG level was significantly related to an LOS >7 days when it was >163 mg/dl (*β* = 1.327, 95% CI: 1.122–1.569). Thus, the outcome definitions were related to the turning point, influencing their association.

**Table 3:** Threshold effect analysis of IG on prolonged LOS or >7 days LOS.

		Prolonged LOS		>7 days LOS	
		*β* (95% CI)	*P*-value	*β* (95% CI)	*P*-value
	IG				
Fitting by standard linear model		1.018 (1.011–1.025)	<0.001	1.020 (1.013–1.027)	<0.001
Fitting by two-piecewise linear model	In turning point (*K*)	*K* = 137 mg/dl		*K* = 163 mg/dl	
	<*K* effect 1	0.989 (0.976–1.001)	0.071	1.001 (0.991–1.011)	0.827
	>*K* effect 2	1.063 (1.040–1.087)	<0.001	1.327 (1.122–1.569)	<0.001
	Effect 2 − 1	1.075 (1.043–1.108)	<0.001	1.325 (1.117–1.573)	0.001
	log-likelihood ratio		<0.001		<0.001

IG, intraoperative glucose; LOS, length of hospital stay; CI, confidence interval.

### 3.3 The clinical value of IG in predicting hospitalization time

**Figure 3:**
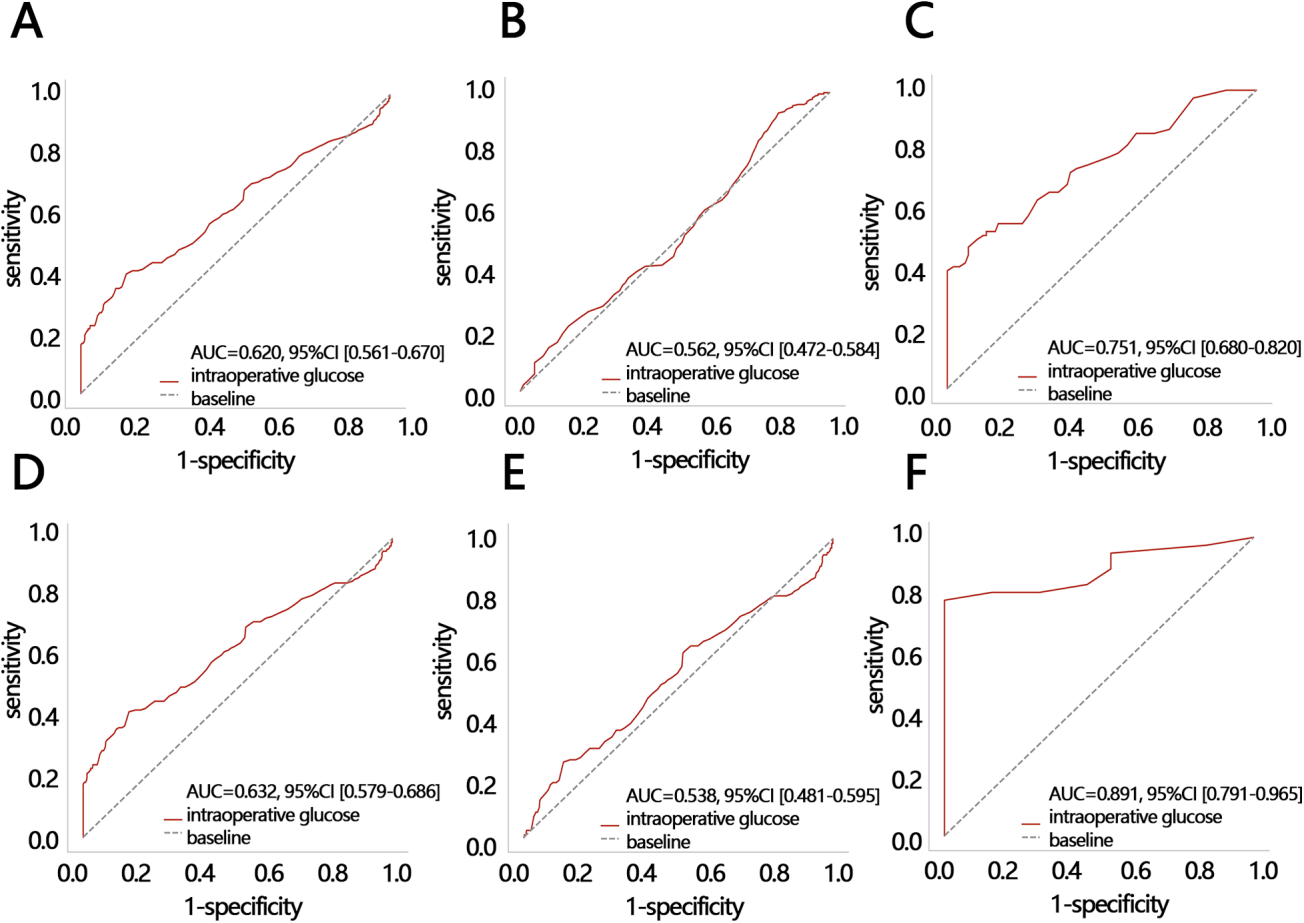
The ROC analyses. The ROC of IG level for predicting a prolonged LOS in **(A)** all participants, **(B)** participants with an IG level <137 mg/dl, and **(C)** participants with an IG level >137 mg/dl. The ROC of IG for predicting an LOS >7 days in **(D)** all participants, **(E)** participants with an IG level <163 mg/dl, and **(F)** participants with an IG level >163 mg/dl. IG, intraoperative glucose; LOS, length of hospital stay; ROC, receiver operating characteristic; AUC, areas under curve; CI, confidence interval.

Due to their significant association, we next assessed the clinical value of IG level in predicting a prolonged length of stay and a 7-day length of stay. We found that IG level had potential value in predicting a prolonged length of hospital stay among all the participants (Figure 3A, AUC = 0.620) and it especially showed favorable predicting performance in participants with an IG level >137 mg/dl (AUC = 0.751) compared with participants with an IG level <137 mg/dl (Figures 3B,C). Uniformly, IG level had a similar function in predicting an LOS of >7 days among all the participants (Figure 3D, AUC = 0.632). It also showed better predicting performance among participants with an IG level >163 mg/dl (Figures 3E,F). Taken together, our results indicated the promising value of IG level in predicting a prolonged length of hospital stay among participants with IG >137 mg/dl or predicting an LOS of >7 days among participants with an IG level >163 mg/dl.

**Figure 4:**
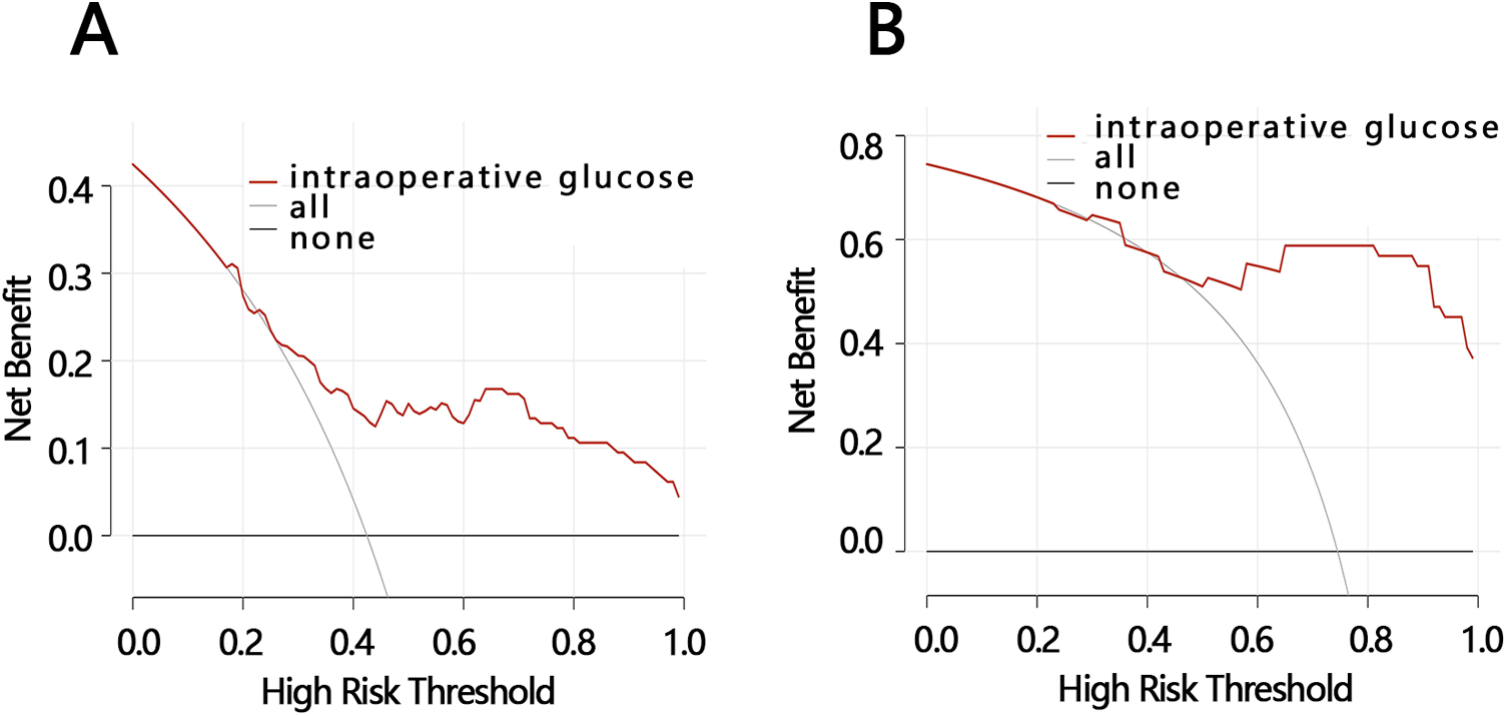
DCA analyses. **(A)** The DCA of IG level for predicting a prolonged LOS in participants with an IG level >137 mg/dl. **(B)** The DCA of IG level for predicting an LOS >7 days in participants with an IG level >163 mg/dl. IG, intraoperative glucose; LOS, length of hospital stay; DCA, decision curve analysis.

Therefore, we further compared the net benefit between participants with an IG level >137 mg/dl and an IG level >163 mg/dl via DCA for the outcome of LOS. The *X*-axis of the DCA represents the threshold probability, which is the probability level at which a patient is predicted/assumed to be positive and would choose to receive treatment/intervention. The *Y*-axis represents net benefit, a metric that takes into account both “benefit” and “harm.” Benefit refers to the advantage gained from treating true-positive patients, while harm refers to the damage caused by treating false-positive patients. The results showed that when the risk threshold was 25%–100%, the highest net benefit was 0.16 for a prolonged LOS as an outcome in participants with an IG level >137 mg/dl (Figure 4A); when the risk threshold was 45%–100%, the highest net benefit was 0.6 for an LOS >7 days as an outcome in participants with an IG level >163 mg/dl (Figure 4B). These results indicated that an LOS >7 days as an outcome in participants with an IG level >163 mg/dl had a better net benefit compared to a prolonged LOS as an outcome in participants with an IG level >137 mg/dl. The above results indicated that IG level can achieve better net benefit in predicting an LOS >7 days if it is >163 mg/dl.

## 4 Discussion

This study included 743 patients with femoral neck fractures, among whom 185 cases (24.90%) experienced a prolonged hospitalization. This rate is slightly lower than the 30.79% reported by Shi et al. (9). The reason for this difference may be attributed to the inclusion of other fracture cases besides femoral neck fractures in Shi et al.'s study, which could lead to differences in both overall case characteristics and individual differences, resulting in inconsistent reported outcomes. Chattaris et al. (10) reported an average LOS of 5.5 days for patients with mainly femoral neck fractures. In this study, the average LOS for patients with femoral neck fractures was 6.08 days, slightly higher than the aforementioned reported results, with the longest hospitalization period reaching 76.9 days. Therefore, it is necessary to continue investigating LOS for patients with femoral neck fractures. LOS is one of the most important indicators of fracture care and is closely related to in-hospital complications, postoperative outcomes, and medical expenses (11). Therefore, identifying factors influencing prolonged hospitalization for femoral neck fracture patients is crucial for targeted interventions and outcome improvement. Although previous studies have reported relevant influencing factors such as age and gender (12), there is still a lack of investigation into intraoperative indicators. MacDonald et al. (13) clearly emphasized the importance of monitoring and managing IG levels in their study. Based on the above, this study primarily explored the potential impact of IG levels on LOS for patients with a femoral neck fracture.

Previous studies on other diseases (14) have found that the higher the glucose level, the greater the risk of an LOS >7 days (OR = 1.45, 95%CI = 1.02–2.05). This indicates an association between glucose levels and length of hospital stay. This study shows that IG levels are associated with length of hospital stay, a prolonged LOS, and an LOS >7 days in a non-linear relationship. This result persists after adjusting for all confounding factors in the model, suggesting that the effect of IG levels on prolonged hospitalization for patients with femoral neck fractures is relatively independent of other factors. The analysis suggests that elevated IG levels trigger the release of inflammatory cytokines, leading to a series of inflammatory reactions, microcirculatory disturbances, and functional impairments, affecting postoperative recovery and thereby prolonging hospitalization (15). Elevated IG levels also increase the risk of postoperative infection, prolonging recovery time and thus increasing hospitalization. Research by Hu et al. (16) indicates that IG levels are an independent influencing factor for postoperative wound infection in fracture surgery. Elevated glucose levels can also increase lactic acid accumulation, downregulate immune function, cause electrolyte disturbances and metabolic abnormalities, and thus affect the postoperative prognosis, increasing hospitalization (17). Threshold effects showed that an IG level >137 mg/dl indicated a significantly increased risk of a prolonged LOS. An IG level >163 mg/dl indicated a significantly increased risk of an LOS >7 days. This was consistent with the study by Cruz (18). Low blood sugar is also independently associated with prolonged hospitalization. However, this study did not show a warning significance when the IG level was <163 mg/dl (or <137 mg/dl), which may be related to factors such as sample size and individual differences in cases.

Further research has found that IG level has clinical value in predicting LOS for patients, but its predictive value is related to the setting of patient clinical outcomes. Specifically, in patients with an IG level >163 mg/dl, using glucose to predict a patient's LOS being >7 days performs best and achieves the optimal clinical net benefit. This model can help identify patients with a higher probability of requiring long hospital stays and assist hospital management in strategically planning bed capacity to reduce overcrowding and underutilization when coordinating surgical volumes. Compared to the model developed by Rodney A. Gabriel based on multiple factors (age, opioid use, metabolic equivalent score, gender, anemia, chronic obstructive pulmonary disease, hypertension, obesity, and primary anesthesia type) to predict no need for a prolonged hospital stay after primary total hip arthroplasty, this model is more concise and efficient [AUC: IG model (0.891) vs. multi-factor model (0.735)] (19).

Our study has two strengths. First, it excluded patients with diabetes and focused on non-diabetic patients with femoral neck fractures, aiming to clarify the impact of IG levels on LOS for this patient group. Second, the study identified the most suitable population for predicting LOS using IG level (patients with IG >163 mg/dl), and the clinical outcome was set as a 7-day LOS, maximizing the clinical predictive value. However, the limitation of this study lies in its retrospective nature as it analyzed data from the MIMIC-IV database, which mainly consists of American patients and does not encompass a global perspective. In addition, the study did not show a causal relationship between IG level and LOS due to it being a retrospective study (20). Therefore, in the follow-up study, we will use a prospective study similar to Cheng et al. (21) to verify our results.

## 5 Conclusion

In summary, elevated IG levels increased the risk of a longer LOS in patients with femoral neck fractures. There was a non-linear relationship between these two variables which was not influenced by other confounding factors. In addition, we found that among patients with IG levels >163 mg/dl, using IG to predict the >7 days LOS had favorable performance and yielded a good clinical net benefit.

## Data Availability

The raw data supporting the conclusions of this article will be made available by the authors, without undue reservation.

## References

[1] Long Y Wang T Xu X Ran G Zhang H Dong Q Risk factors and outcomes of extended length of stay in older adults with intertrochanteric fracture surgery: a retrospective cohort study of 2132 patients. J Clin Med. (2022) 11(24):7366. 10.3390/jcm11247366 36555982 PMC9784786

[2] Manosroi W Koetsuk L Phinyo P Danpanichkul P Atthakomol P. Predictive model for prolonged length of hospital stay in patients with osteoporotic femoral neck fracture: a 5-year retrospective study. Front Med (Lausanne). (2022) 9:1106312. 10.3389/fmed.2022.1106312 36714117 PMC9874094

[3] Schneider AM Mucharraz C Denyer S Brown NM. Prolonged hospital stay after arthroplasty for geriatric femoral neck fractures is associated with increased early mortality risk after discharge. J Clin Orthop Trauma. (2022) 26(1):101785. 10.1016/j.jcot.2022.101785 35211374 PMC8844821

[4] Ping Li HC Sun Y. Influencing factors of postoperative stress hyperglycemia in orthopedic patients. Chin Nurs Res. (2021) 35(3):561–2. 10.12102/j.issn.1009-6493.2021.03.044

[5] Meng Y Fu M Zhao Y Zhang Y Wang Z. Interpretation of ‘2020 edition of expert consensus on perioperative blood glucose management’. J Hebei Med Univ. (2022) 43(1):1–6, 11. 10.3969/j.issn.1007-3205.2022.01.001

[6] Price CE Fanelli JE Aloi JA Anzola SC Vishneski SR Saha AK Feasibility of intraoperative continuous glucose monitoring: an observational study in general surgery patients. J Clin Anesth. (2023) 87:111090. 10.1016/j.jclinane.2023.111090 36913777

[7] Klemt C Tirumala V Barghi A Cohen-Levy WB Robinson MG Kwon YM. Artificial intelligence algorithms accurately predict prolonged length of stay following revision total knee arthroplasty. Knee Surg Sports Traumatol Arthrosc. (2022) 30(8):2556–64. 10.1007/s00167-022-06894-8 35099600

[8] Lu CX Huang ZB Chen XM Wu XD. Predicting prolonged postoperative length of stay risk in patients undergoing lumbar fusion surgery: development and assessment of a novel predictive nomogram. Front Surg. (2022) 9:925354. 10.3389/fsurg.2022.925354 36051703 PMC9426777

[9] Shi HZ Zheng HW Zhang J Zhao WY Wang HY Yang X Risk factors analysis and prediction model construction of prolonged length of stay in oldest-old patients with hip fracture after surgery. J Nurs Sci. (2023) 38(5):20–4. 10.3870/j.issn.1001-4152.2023.05.020

[10] Chattaris T Chahal K Berry SD. Factors besides frailty index affect length of stay in older patients with hip fractures. Osteoporos Int. (2023) 34(8):1493–4. 10.1007/s00198-023-06798-4 37246196 PMC10225280

[11] Horton I Bourget-Murray J Buth O Backman C Green M Papp S Delayed mobilization following admission for hip fracture is associated with increased morbidity and length of hospital stay. Can J Surg. (2023) 66(4):E432–E8. 10.1503/cjs.006822 37643796 PMC10473868

[12] Schneider AM Denyer S Brown NM. Risk factors associated with extended length of hospital stay after geriatric hip fracture. J Am Acad Orthop Surg Glob Res Rev. (2021) 5(5):e2100073. 10.5435/JAAOSGlobal-D-21-00073 33945514 PMC8099404

[13] MacDonald DB Mackin MJ. Intraoperative glucose management: when to monitor and who to treat? Can J Anaesth. (2023) 70(2):177–82. 10.1007/s12630-022-02358-2 36450942

[14] Wang D Lu J Zhang P Hu Z Shi Y. Relationship between blood glucose levels and length of hospital stay in patients with acute pancreatitis: an analysis of MIMIC-III database. Clin Transl Sci. (2023) 16(2):246–57. 10.1111/cts.13445 36350303 PMC9926064

[15] Zhong H Wang B Wang D Liu Z Xing C Wu Y The application of machine learning algorithms in predicting the length of stay following femoral neck fracture. Int J Med Inform. (2021) 155:104572. 10.1016/j.ijmedinf.2021.104572 34547625

[16] Hu Q Zhao Y Sun B Qi W Shi P. Surgical site infection following operative treatment of open fracture: incidence and prognostic risk factors. Int Wound J. (2020) 17(3):708–15. 10.1111/iwj.13330 32068337 PMC7949428

[17] Knaak C Wollersheim T Morgeli R Spies C Vorderwulbecke G Windmann V Risk factors of intraoperative dysglycemia in elderly surgical patients. Int J Med Sci. (2019) 16(5):665–74. 10.7150/ijms.32971 31217734 PMC6566747

[18] Cruz P. Inpatient hypoglycemia: the challenge remains. J Diabetes Sci Technol. (2020) 14(3):560–6. 10.1177/1932296820918540 32389071 PMC7576945

[19] Gabriel RA Sharma BS Doan CN Jiang X Schmidt UH Vaida F. A predictive model for determining patients not requiring prolonged hospital length of stay after elective primary total hip arthroplasty. Anesth Analg. (2019) 129(1):43–50. 10.1213/ANE.0000000000003798 30234533

[20] Shi L Le K Qi H Feng Y Zhou L Wang J The safety and efficacy of delayed surgery by simulating clinical progression of observable papillary thyroid microcarcinoma: a retrospective analysis of 524 patients from a single medical center. Front Oncol. (2023) 13:1046014. 10.3389/fonc.2023.1046014 37881490 PMC10597687

[21] Cheng J Lao Y Chen X Qiao X Sui W Gong X Dynamic nomogram for subsyndromal delirium in adult intensive care unit: a prospective cohort study. Neuropsychiatr Dis Treat. (2023) 19:2535–48. 10.2147/NDT.S432776 38029051 PMC10676691

